# A single-center, retrospective review of robot-assisted laparoscopic prostatectomy with and without cryopreserved umbilical cord allograft in improving continence recovery

**DOI:** 10.1007/s11701-019-00972-9

**Published:** 2019-05-31

**Authors:** Mutahar Ahmed, Michael Esposito, Gregory Lovallo

**Affiliations:** NJ Center for Prostate Cancer and Urology, 255 W Spring Valley Ave #101, Maywood, NJ 07607 USA

**Keywords:** Urinary incontinence, Prostatectomy, Biological dressing, Umbilical cord allograft, Nerve wrap, Neurovascular bundle

## Abstract

The objective of this study was to evaluate the safety and effectiveness of cryopreserved umbilical cord (UC) allograft as a nerve wrap around the neurovascular bundle (NVB) in accelerating return to continence after radical prostatectomy. A single-center, retrospective study was performed on 200 patients who underwent bilateral, nerve-sparing robot-assisted radical prostatectomy (RARP) with and without placement of UC around the NVBs (*n* = 100/group). Patients were excluded if they had previous simple or transurethral prostatectomy or history of pelvic radiation. Post-operative continence, defined as 0 or 1 safety pad, was analyzed between groups at 1, 3, 6, and 12 months. Complications, biochemical recurrence and adverse events were assessed to determine safety. Patients who underwent RARP with UC were significantly more likely to be continent at 1 month (65% vs. 44%, *p* = 0.018), 3 months (83% vs. 70%, *p* = 0.03), and 12 months (97% vs. 87%, *p* = 0.009). Sample stratification revealed that UC is beneficial for obese patients and those > 60 years, both of which are high risk for post-RARP incontinence. Biochemical failure was noted in 2 (UC) and 4 (control) patients. No adverse events or complications related to UC were observed. The results suggest that UC allograft is safe and accelerates continence recovery in post-RARP patients. Prospective, randomized trials are warranted.

## Introduction

Prostate cancer is the most common cancer diagnosis among men, with more than 160,000 new cases in the United States each year [1]. Radical prostatectomy (RP) is the recommended, front-line treatment approach for patients with clinically localized prostate cancer and greater than 10-year life expectancy [2]. There are several surgical approaches for RP, with the majority of cases conducted via the minimally invasive da Vinci robotic-assisted surgical system [3, 4]. This technology provides surgeons with superior visualization, enhanced dexterity and greater precision, and as a result, improves surgical and functional outcomes. Despite these operative advances, urinary incontinence remains a problem for at least 50% of men who undergo robot-assisted prostatectomy [5–8]. Consequently, these men may have a poor quality of life and report anxiety, fear and embarrassment [8], as well as loss of sense of control, depression and decreased social interactions [6]. Unfortunately, post-prostatectomy incontinence is expected to rise due to the increasing number of procedures performed which increases burden on society [9].

Urethral sphincter incompetence is generally considered as the most important contributing factor to post-RP incontinence and is likely a result of damage to supporting structures and nerves rather than damage to the sphincter itself [10, 11]. It has been found that the neurovascular bundle (NVB) directly innervates the membranous urethra [12], and intraoperative stimulation of the NVB significantly increases urethral pressure [13]. These findings suggest that NVB damage affects the continence mechanism, and preservation leads to earlier recovery of continence following RP [14–21]. While NVB preservation minimizes incontinence, it is often difficult to completely mitigate NVB manipulation as anatomic studies show a plate-like formation of nerves rather than the traditional bundle [10]. Furthermore, dissection, traction, and surgical insult can elicit inflammation, mitigating the healing process and impacting continence after prostatectomy.

Amniotic and umbilical cord (UC) tissues have been used in a wide variety of clinical applications to reduce inflammation and promote regenerative healing including ocular surface reconstruction [22, 23], tendon repair [24, 25], wound healing [26–33], and burns [34–37]. Additionally, amniotic membrane has been shown to support nerve regeneration [38–40], with evidence of increased axons possessing myelin sheaths of normal thickness, as well as, less inter-axonal fibrosis [38]. While the clinical use of UC is well documented, its use in reducing inflammation and promoting regenerative healing in the NVB is relatively new. Several recent studies have demonstrated enhanced return to potency using dehydrated human amnion/chorion membrane [41–43], with only one of them reporting continence outcomes [41]. While the short-term results are encouraging, no studies have assessed continence outcomes past 2 months. The aim of this study was to assess the use of cryopreserved UC allograft as a nerve wrap during robot-assisted radical prostatectomy (RARP) in accelerating return to continence up to 1-year post-op.

## Materials and methods

Full institutional board approval and waiver of informed consent was granted for this study. A retrospective medical chart review was conducted on patients who underwent bilateral nerve-sparing RARP with and without cryopreserved UC allograft (CLARIX CORD 1K, Amniox Medical Inc., Miami, FL, USA) from January 2015 to September 2017. Patients were eligible for inclusion in this study if they had at least 3 months of follow-up data. Patients were excluded if they had previous history of pelvic radiation or prostatectomy or NVB damage during surgery.

### Treatment procedures

All RARP procedures were performed at Hackensack Meridian Health Hackensack University Medical Center (Hacensack, NJ, USA) and all patients were followed up at the New Jersey Center for Prostate Cancer and Urology (Maywood, NJ, USA) by three surgeons using the standard trans-peritoneal six-port technique with the da Vinci surgical system (Intuitive Surgical, Sunnyvale, CA, USA). Bilateral, athermal nerve-sparing RARP was performed on each patient, with bladder neck reconstruction, an anterior suspension stitch, and posterior reconstruction. A 6 × 3.0 cm UC allograft (CLARIX CORD 1K, Amniox Medical, Miami, FL, USA) was cut into two longitudinal pieces (1.5 cm in width) and placed circumferentially around each NVB as a nerve wrap through an assistant port.

### Outcome measures

Data collected from the medical charts included demographic information, significant medical history including co-morbidities, prostate size, blood loss, perineural invasion, positive surgical margins, PSA levels, Gleason score, and clinical stage. The primary endpoint was the proportion of men with return of urinary continence at follow-up. Continence was defined as use of no or one safety pad. The secondary endpoint was the safety of UC for use as NVB wrap during NS-RARP by assessing both treatment related and treatment emergent adverse events through review of physical exams and assessments. Adverse events were further classified as procedure related or product related.

### Statistical analysis

Post-operative outcomes were analyzed between groups using the Student’s *t* test for continuous factors and the Chi-Square or Fisher’s exact test for categorical factors. To identify and adjust for factors that could influence continence rates at each follow-up, multiple regression analysis was implemented. All statistical analyses were performed using SPSS v. 20.0 (IBM SPSS Statistics, Chicago, IL, USA). A *p* value < 0.05 was used to determine statistical significance.

## Results

A total of 200 patients who underwent bilateral nerve-sparing RARP with and without cryopreserved UC (*n* = 100/group) met the eligibility criteria and were included for retrospective analysis. Demographics, pre-operative characteristics (Table 1), and intraoperative outcomes (Table 2) were comparable between the UC and control group. The mean age in the UC group was 61.9 ± 7.1 years, while the control group was 59.6 ± 7.0 years (*p* = 0.05). Additionally, the BMI was 28.5 ± 4.6 kg/m^2^ in the UC group and 29.2 ± 5.2 kg/m^2^ in the control group (*p* = 0.39). Clinical stage, surgical margins, prostate size, and PSA levels did not differ between groups.

**Table 1:** Patient demographics and preoperative characteristics

	Control group	UC group	*p* value
Age (years)	59.62 ± 6.97 60.0 (41, 74)	61.91 ± 7.08 61.5 (44, 77)	0.05
BMI (kg/m^2^)	29.15 ± 5.17 28.27 (20.1, 43.8)	28.53 ± 4.61 27.98 (18.5, 42.7)	0.39
Underweight	0 (0%)	1 (1%)	
Normal	20 (20%)	16 (16%)	
Overweight	42 (42%)	41 (41%)	
Obese	38 (38%)	27 (27%)	
Unknown	0 (0%)	15 (15%)	
Comorbidities, *n* (%)			
Hypertension	39 (39%)	39 (39%)	1.0
Diabetes	13 (13%)	7 (7%)	0.16
Hypercholesteremic	19 (19%)	19 (19%)	1.0
Smoking history			0.13
Yes	11 (11%)	20 (20%)	
No	61 (61%)	52 (52%)	
Former	28 (28%)	21 (21%)	
Unknown	0 (0%)	7 (7%)	
PSA (ng/mL)	7.94 ± 8.88 5.4 (0.33, 65.0)	6.60 ± 4.55 5.4 (0.40, 23.5)	0.19
Gleason score, *n* (%)			0.001
≤ 6	37 (37%)	14 (14%)	
7	56 (56%)	76 (76%)	
≥ 8	7 (7%)	10 (10%)	
Clinical stage, *n* (%)			0.52
pT1c	0 (0%)	1 (1%)	
pT2a	6 (6%)	3 (3%)	
pT2b	0 (0%)	1 (1%)	
pT2c	70 (70%)	64 (64%)	
pT3a	13 (13%)	18 (18%)	
pT3b	11 (11%)	13 (13%)	

*BMI* body mass index, *PSA* prostate specific antigen

^a^Data presented as mean ± SD, median (min, max) or number (percent) as indicated

**Table 2:** Comparison of intraoperative outcomes

	Control group	UC group	*p* value
Prostate size (g)	48.74 ± 17.15 45 (22, 132)	51.57 ± 16.98 48 (16, 119)	0.24
Prostate volume (cm^3^)	75.76 ± 38.66 65.6 (17.8, 235.1)	77.23 ± 37.92 70.9 (15, 252)	0.79
Perineural invasion, *n* (%)	57 (57%)	71 (71%)	0.04
Blood loss, *n* (%)			0.01
< 50 mL	9 (9%)	23 (23%)	
50 mL	19 (19%)	16 (16%)	
75 mL	0 (0%)	3 (3%)	
100 mL	38 (38%)	24 (24%)	
150 mL	11 (11%)	3 (3%)	
> 150 mL	6 (6%)	5 (5%)	
Unknown	17 (17%)	26 (26%)	
Surgical margins, *n* (%)			0.21
Positive	24 (24%)	24 (24%)	
Negative	75 (75%)	49 (49%)	
Unknown	1 (1%)	27 (27%)	

^a^Data presented as mean ± SD, median (min, max) or number (percent) as indicated

**Fig. 1:**
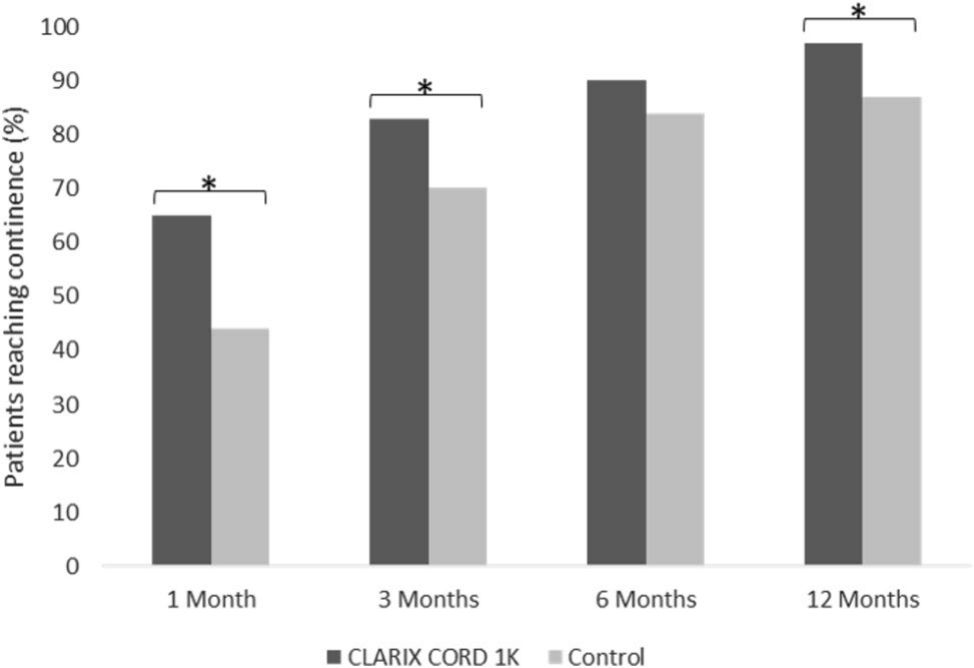
Continence recovery outcomes at 1, 3, 6, and 12 months post-RARP. * Indicates 5% significance (*p* < 0.05)

Continence recovery rates at 1, 3, 6, and 12 months were significantly better for patients receiving UC when compared to the control group at all points in time, except at 6 months: 65% (42/65) vs. 44% (31/70) at 1 month (*p* = 0.018), 83% (83/100) vs. 70% (70/100) at 3 months (*p* = 0.03), 90% (90/100) vs. 84% (84/100) at 6 months (*p* = 0.21), and 97% (97/100) vs. 87% (87/100) at 12 months (*p* = 0.009) (Fig. 1). When defining continence as use of zero pads, continence recovery rates were significantly better for patients receiving UC at 1, 3, 6, and 12 months compared to the control group at all time points: 55% (36/65) vs. 30% (21/70) at 1 month (*p* = 0.003), 68% (68/100) vs. 52% (52/100) at 3 months (*p* = 0.021), 84% (84/100) vs. 64% (64/100) at 6 months (*p* = 0.001), and 90% (90/100) vs. 80% (80/100) at 12 months (*p* = 0.048).

To identify and adjust for factors that could influence continence at each follow-up, binary logistic regression analysis was implemented. The factors included in each model were treatment, age, BMI, diabetes, hypertension, Gleason score, perineural invasion, blood loss, and prostate size. After controlling for these factors, only treatment and age were significant predictors of continence outcomes at 1 month (*p* = 0.02 and 0.006, respectively), 3 months (*p* = 0.032 and 0.001, resp.) and 12 months post-RARP (*p* = 0.005 and 0.001, resp.). At 6 months, only age was predictive of continence (*p* = 0.001).

**Fig. 2:**
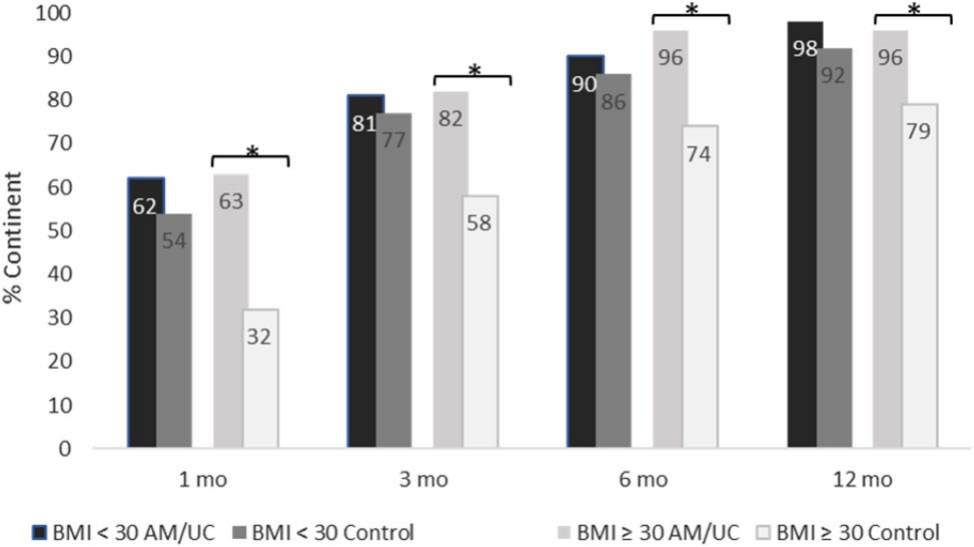
Continence recovery outcomes stratified by BMI. * Indicates 5% significance (*p* < 0.05)

**Fig. 3:**
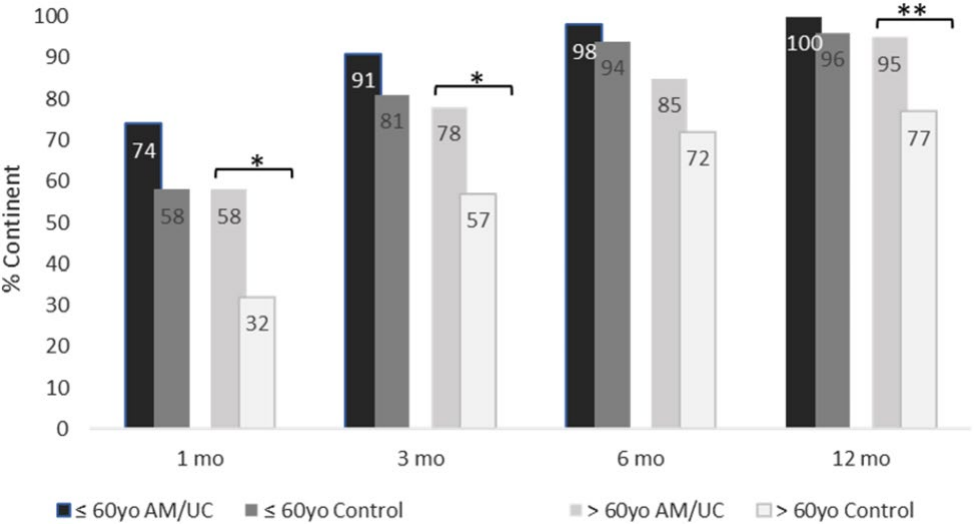
Continence recovery outcomes stratified by age. * Indicates 5% significance (*p* < 0.05) and ** indicates 1% significance (*p* < 0.01)

The study sample was further stratified into two groups according to BMI and age, and the continence recovery rates at 1, 3, 6, and 12 months were compared between the UC and control group. For patients ≥ 30 kg/m^2^ (*n* = 65), continence recovery rates were significantly better in the UC group than the control group at all time points (*p* < 0.05), while there was no significant difference between groups for patients < 30 kg/m^2^ (*n* = 120) (Fig. 2). In addition, for patients > 60 years old (*n* = 105), the UC group was significantly more continent than the control group at 1, 3, and 12 months (*p* < 0.05); whereas, continence did not significantly differ between groups in patients ≤ 60 years (*n* = 95) at any time point (Fig. 3).

Safety was assessed through review of physical exams and assessments for both treatment related and treatment emergent adverse events. No complications or adverse events related to UC were observed throughout the duration of the study. In addition, biochemical failure was noted in 2 (UC) and 4 (control) patients and a nonsignificant improvement in potency (SHIM > 16) was noted in patients who received UC.

## Discussion

Despite recent advances in operative technique, there remains a convalescent period characterized by urinary incontinence even when the NVBs are well preserved [44–46]. This delay in continence recovery is believed to be a result of dissection or traction injury to the NVB and supporting structures which induces an inflammatory response [11, 47]. In this retrospective review, we assessed the clinical effectiveness and safety of cryopreserved UC as a NVB wrap in facilitating continence recovery in patients who underwent bilateral, NS-RARP due to the known anti-inflammatory actions of the UC. Our results showed that there was a significantly higher percentage of patients who became continent in the UC vs. control group at 1 month (65% vs. 44%), 3 months (83% vs. 70%,), and 12 months post-RARP (97% vs. 87%). After controlling for possible covariates influencing continence outcomes, UC and age were found to have a significant effect on continence recovery at 1, 3, and 12 months. Moreover, we found that UC significantly benefits patients with a BMI ≥ 30 kg/m^2^ or patients > 60 years old. These findings are especially promising given obesity [48–51] and old age [50, 52–56] are significant risk factors of post-RP incontinence, specifically at 6- and 12 months [57].

Other attempts have been made to reduce post-surgical inflammation after RARP. Some studies evaluated the induction of regional hypothermia via an endorectal cooling balloon; however, a recent randomized control trial demonstrated no significant benefit for continence recovery [58]. In addition, other studies have assessed the use of dehydrated AM tissue around the NVB in accelerating return to potency; however, only one study has reported continence outcomes [41]. In that study, Patel et al. found that the mean time to continence was shorter in the dHAM group by 0.62 months (*p* = 0.03); however, there was no significant difference in continence outcomes between the dHAM and control group at the 2-month follow-up (81% vs. 74%, *p* = 0.37). It is possible that the lack of significant findings is due to the surgical technique or method of AM preservation. While cryopreserved tissues retain their native architecture and biological components relevant to fresh UC tissue [59, 60], dehydrated AM tissues are structurally compromised and almost entirely lack key biological signaling complex, HC-HA/PTX3 [59].

The overall improvement in continence observed in this study may be explained by the anti-inflammatory and regenerative properties inherent to UC tissues [61–63]. UC induces apoptosis of neutrophils [64, 65], monocytes, and macrophages [66] and reduces infiltration of neutrophils [64, 65], macrophages [67, 68], and lymphocytes [69]. Additionally, UC is rich in cytokines and neurotrophic factors, particularly nerve growth factor, which plays an important role in nerve regeneration and epithelial healing [70–73]. One key biological modulator present in cryopreserved UC tissues, HC-HA/PTX3, upregulates IL-10, downregulates IL-12, and polarizes macrophages toward the M2 phenotype, all of which play an integral role in the healing process [61–63]. Together, these conditions provide an optimal environment for healing and facilitate recovery of the NVB post-RARP.

While our data are encouraging, this study is limited by its lack of prospective randomization and potential placebo bias. Patients treated with UC were also predominantly treated after April 2016 compared to control patients treated prior to them; however, the surgical technique was similar during this period. Several pre-operative characteristics significantly differed between groups; however, the treatment group represented a higher risk sample as age and Gleason scores were more advanced. At our practice, UC is used for patients undergoing only bilateral nerve-sparing RARP, with < T4 prostate cancer, without erectile dysfunction pre-op, and without neurogenic bladders. Future health economic studies are needed to determine cost and benefit analysis of using the UC (at a cost of approximately $2500). Prospective, randomized clinical trials with validated questionnaires are necessary to further ascertain the effect of UC on urinary incontinence following NS-RARP.

## Conclusion

The results of this retrospective study suggest that UC can be safely used to facilitate return to continence following NS-RARP. Prospective, randomized trials are warranted to further evaluate the potential benefit of UC.
